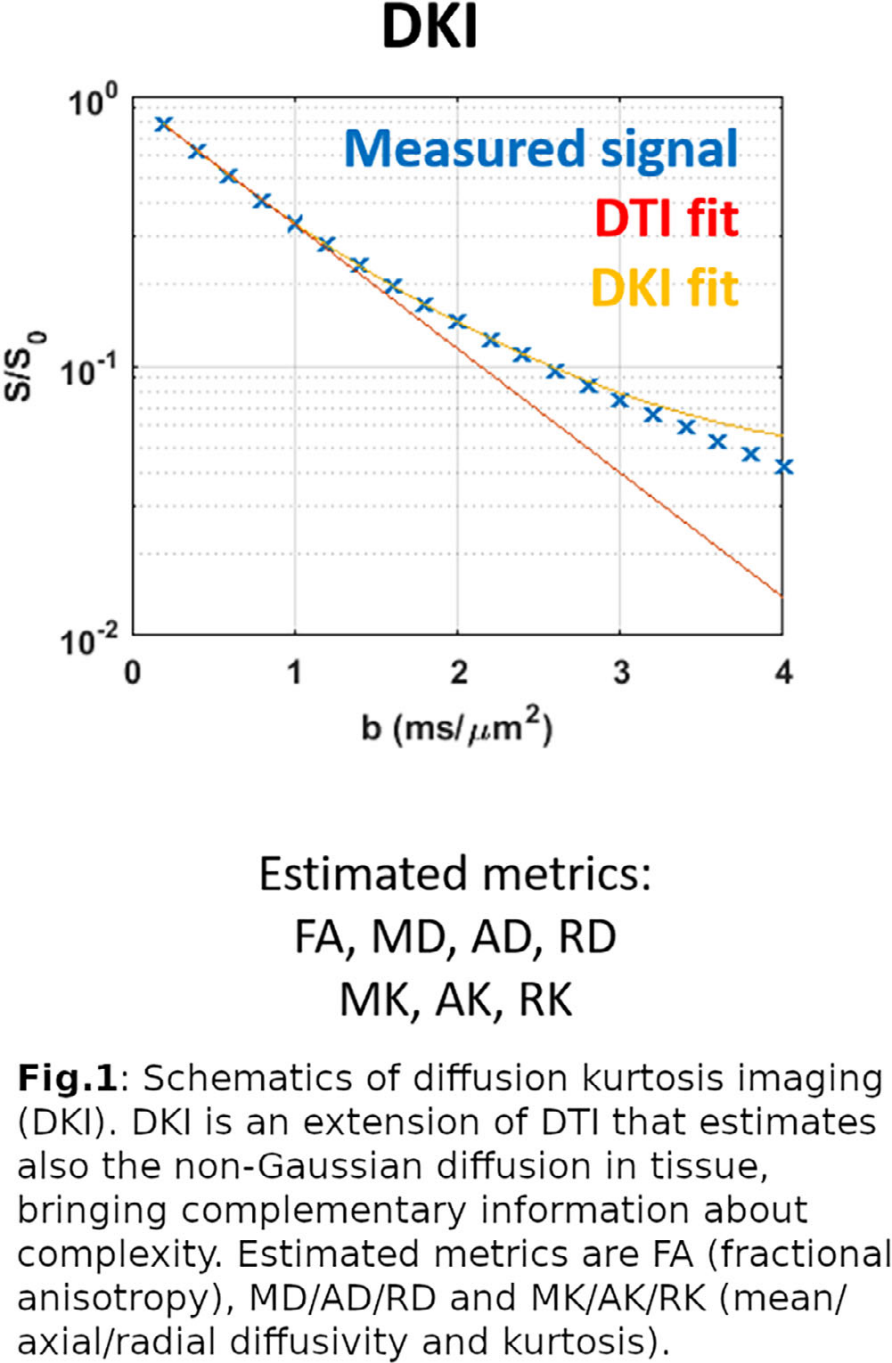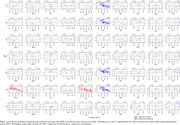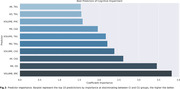# Diffusion Kurtosis Imaging provide complementary information over structural MRI in the assessment of hippocampal subfields in patients with cognitive decline

**DOI:** 10.1002/alz.091263

**Published:** 2025-01-09

**Authors:** Tommaso Pavan, Michela Pievani, Federica Ribaldi, Jorge Jovicich, Valentina Garibotto, Giovanni B. Frisoni, Ileana O Jelescu

**Affiliations:** ^1^ Department of Radiology, Lausanne University Hospital (CHUV), Lausanne Switzerland; ^2^ University of Lausanne (UNIL), Lausanne Switzerland; ^3^ Laboratory of Alzheimer's Neuroimaging and Epidemiology ‐ LANE, IRCCS Istituto Centro San Giovanni di Dio Fatebenefratelli, Brescia Italy; ^4^ Geneva Memory Center, Department of Rehabilitation and Geriatrics, Geneva University Hospitals, Geneva Switzerland; ^5^ Laboratory of Neuroimaging of Aging, University of Geneva, Geneve Switzerland; ^6^ Magnetic Resonance Imaging Laboratory Center for Mind/Brain Sciences, University of Trento, Mattarello Italy; ^7^ Division of Nuclear Medicine and Molecular Imaging, Geneva University Hospitals, Geneva Switzerland; ^8^ Laboratory of Neuroimaging and Innovative Molecular Tracers (NIMTlab), Geneva University Neurocenter and Faculty of Medicine, University of Geneva, Geneve Switzerland; ^9^ Laboratory of Neuroimaging of Aging, University of Geneva, Geneva Switzerland

## Abstract

**Background:**

Hippocampal atrophy is an established biomarker of neurodegeneration in Alzheimer's disease, affecting specific subfields (De Flores, La Joie and Chételat, 2015). In this study, we used 7T MRI and advanced diffusion MRI (dMRI) to investigate the relationship between hippocampal subfield volumes and microstructure and assess their sensitivity to cognitive impairment.

**Method:**

Seventeen cognitively impaired (CI; age: 69±8, M/F: 12/5, MMSE: 28) and 22 cognitively unimpaired subjects (CU; age: 62±10, M/F: 6/16) were recruited in the context of the COSCODE project (Ribaldi et al., 2021). MRI was performed at 7 Tesla, including a high resolution T2w FSE sequence for hippocampal segmentation and multi‐shell diffusion MRI sequence for kurtosis tensor imaging (DKI, Jensen and Helpern, 2010) fitting. Hippocampal subfields were segmented using Automated Segmentation of Hippocampal Subfields (ASHS) package. Microstructure analysis included DTI and DKI (Figure 1). Mean values for each diffusion metric were calculated in subfields of the ASHS atlas (Xie et al. 2016). Subfield volumes were normalised by the total intracranial volume while dMRI metrics were residualized by age via a linear regression trained only on CU and then correlated to the volumetric data across within each group (statistical significance: p<0.05). Lasso logistic regression was used to identify the metrics that better discriminated CI from CU.

**Result:**

Few hippocampal subfields’ volumes correlated with dMRI metrics (Figure 2). In the CU group, DG volume showed negative associations with diffusion metrics, primarily RD (r:‐0.59, p=0.007), MD(r:‐0.56, p=0.012), MK(r:‐0.46, p=0.043) and AK(r:‐0.62, p=0.005). In the CI group, negative correlations between BA35 (r:‐0.70, p=0.008) and CA3 (r:‐0.57, p=0.041) volumes were found with AK. When discriminating CI from CU, the logistic regression found the ERC volume as the most important metric, followed by the MK of the DG, and AK of CA2 (Figure 3).

**Conclusion:**

The disconnection between dMRI metrics and volumetric measures and the selection of DKI metrics in discriminating CI from CU participants suggest that DKI metrics may act as complementary biomarkers to subfield volumes for early detection of cognitive impairment.